# Mortality risk factors in patients with bloodstream infections due to multidrug-resistant Gram-negative bacilli

**DOI:** 10.3389/fmed.2025.1693317

**Published:** 2026-01-12

**Authors:** Ana Luisa Corona-Nakamura, Martha Judith Arias-Merino, Sussan Alely Urbina-Rosas, Martha Elena Vázquez-Arias, Juan Fernando Corona-Macías, Eduardo González-Espinoza, Jorge Andrade-Sierra, Arnulfo Hernán Nava-Zavala, Luis Humberto Govea-Camacho

**Affiliations:** 1 Division of Medicine, National Western Medical Center, Mexican Social Security Institute (IMSS), Guadalajara, Jalisco, Mexico; 2 Western Clinical Research Center, Zapopan, Jalisco, Mexico; 3 Health Sciences University Center, University of Guadalajara, Guadalajara, Jalisco, Mexico; 4 Divisions of Nephrology and Transplantation, National Western Medical Center, Mexican Social Security Institute (IMSS), Guadalajara, Jalisco, Mexico; 5 Department of Physiology, University Center for Health Sciences, University of Guadalajara, Guadalajara, Jalisco, Mexico; 6 School of Medicine International Program, Autonomous University of Guadalajara, Zapopan, Jalisco, Mexico; 7 Department of Immunology and Rheumatology of the Western General Hospital, Jalisco Ministry of Health, Zapopan, Jalisco, Mexico; 8 Epidemiological and Health Services Research Unit, National Western Medical Center, Mexican Social Security Institute (IMSS), Guadalajara, Jalisco, Mexico; 9 Surgery Division, National Western Medical Center, Mexican Social Security Institute (IMSS), Guadalajara, Jalisco, Mexico

**Keywords:** bloodstream infections, multidrug-resistant Gram-negative bacilli, mortality, carbapenem-resistant *Acinetobacter baumannii*, ineffective empirical treatment

## Abstract

**Introduction:**

This study aimed to analyze risk factors for mortality in hospitalized patients with bloodstream infections caused by multidrug-resistant Gram-negative bacilli in a retrospective cohort (January–December 2022).

**Methods:**

Hospitalized patients with positive monomicrobial blood cultures for GNB (from central venous catheters and peripheral venipuncture) were included. Medical records and blood culture isolates were reviewed. The primary endpoint was all-cause mortality at ≤ 30 days. Risk factor analysis was performed using univariate models, survival curves (Cox regression), and an adjusted Cox proportional hazards model.

**Results:**

A total of 126 patients with Gram-negative bacillus bloodstream infection were included, 36 of them died within ≤ 30 days, representing a mortality rate of 28.6%. Of these deaths, 32/36 (88.9%) were due to carbapenem-resistant bacilli. The most frequently isolated gram-negative bacilli were: *Acinetobacter baumannii, Pseudomonas aeruginosa*, and *Klebsiella pneumoniae*. According to the univariate analysis, mortality was 13.2 times higher (95% CI 4.3–40.5; *p* = 0.000) in patients with carbapenem-resistant bacilli and 4.2 times higher (95% CI 1.8–9.6; *p* = 0.001) in those with carbapenem-resistant *A. baumannii*. The main factors associated with all-cause mortality within ≤ 30 days were: age ≥46 years, infection with carbapenem-resistant bacilli, ineffective empirical treatment, and septic shock.

**Discussion:**

Having received ineffective empirical treatment was an independent predictor of mortality, with a hazard ratio (HR) of 10.2 (95% CI: 2.6–39.9; *p* = 0.001). Mortality due to bloodstream infection was related with a high frequency of patients with isolated infection by carbapenem-resistant gram-negative bacilli, mainly *A. baumannii* (CRAB).

## Introduction

WHO estimates that 4.95 million deaths worldwide in 2019 could be associated with patient infections caused by pathogens resistant to antibacterial drugs, affecting mainly low- and middle-income countries (1). Among nosocomial infections, bloodstream infections (BSIs) caused by multidrug-resistant Gram-negative bacilli (MDR-GNB) have become a significant focus of study, given their high morbidity and mortality and economic burden, which directly affect the provision of health services (2–7).

BSI is a pathological entity caused by the dissemination of bacteria into the bloodstream, normally sterile, characterized by fever or hypothermia, hypotension, tachycardia, altered perfusion, tachypnea, and altered mental status, which can potentially cause septic shock. BSIs can be acquired in the community or in the hospital setting (infections acquired at least 48 h after hospitalization and at least 48 h after device insertion). In the hospital setting, they have been classified as primary BSIs associated with central venous catheters (CVCs) and secondary BSIs related to other sources of nosocomial infection (5, 8).

Patients hospitalized with BSI are exposed to a wide variety of heterogeneous and changing pathogens (mainly MDR) and generally have a wide range of severe clinical syndromes. Knowledge of this variety of pathogens is of great importance for its usefulness in hospital epidemiological research and surveillance in the process of improving medical care and optimizing treatments. In highly developed countries, the most frequently reported pathogens are *S. aureus* ( ≤ 30-day case-fatality rate: 21%), *E. coli, Klebsiella spp*., *P. aeruginosa, Enterococcus, Streptococcus*, and coagulase-negative S*taphylococcus*. In Latin America, GNB pathogens are more frequent, with reports of up to 70.6% for the *A. baumannii-calcoaceticus complex*, 28.1% for carbapenem-resistant *Enterobacterales* (CRE), and 26.3% for *P. aeruginosa* (9–11).

Abdel Hadi et al. reported, in a retrospective cohort study, a prevalence of 13% for MDR-GNB bacilli, with higher isolation rates of *E. coli* (62.7%), *K. pneumoniae* (20.4%), *Salmonella species* (6.6%), and *P. aeruginosa* (5.3%). Likewise, they report as risk factors having prolonged hospital stays, presenting multiple comorbidities, having received previous antibiotic treatment, and requiring admission to intensive care units (4).

A mortality of 20%−39% has been reported in BSI due to *A. baumannii* with an antimicrobial resistance of 86%. The survival of multi-resistant strains of *A. baumannii, K. pneumoniae*, and *E. coli* is due to a capsule that protects them from opsonization (5).

At the local and global levels, knowledge and monitoring of microbiologica**l** resistance patterns, particularly among patients with BSI due to MDR-GNB, have a direct impact on health services. Therefore, it is necessary to conduct analytical studies that correlate microbiological isolation and bacterial resistance findings with patient demographics, comorbidities, conditions upon admission, conditions during hospitalization, and other risk factors associated with poor patient outcomes (12).

A previous retrospective cohort study in our hospital observed a mortality rate of 78.2% in patients with ventriculitis due to MDR-GNB associated with an external ventricular drain. *A. baumannii, K. pneumoniae*, and *P. aeruginosa* were the most frequently isolated bacilli. High resistance to carbapenems was observed in *A. baumannii* (91.3%) and *P. aeruginosa* (80.0%) (13). *A. baumannii, P. aeruginosa*, and *K. pneumoniae* were also the most frequently isolated bacilli in the present study, so we consider them potentially present in our hospital healthcare settings.

Thus, the objective of this study was to analyze the risk factors associated with mortality in patients hospitalized for BSI due to MDR-GNB in a retrospective cohort selected from January–December 2022.

## Methodology

### Study design and participants

A retrospective cohort study was conducted among adult patients hospitalized at the National Medical Center of the West, Instituto Mexicano del Seguro Social (January–December 2022), with BSI due to MDR-GNB.

All physical and electronic records of consecutive eligible hospitalized patients with suspected bacteremia in 2022 were reviewed, as were the total blood cultures performed, regardless of results or isolation type. Microbiological results of the isolates were correlated with data from patients suspected of having BSI.

Suspected cases of BSI were defined as those presenting at least two of the following signs and symptoms: fever (above 38.0 °C) or hypothermia (below 36.0 °C), chills, and hypotension (5). The following variables were included in the description and analysis of the selected cohort: age and sex; conditions and comorbidities at admission; conditions during hospitalization (hospitalization service: surgical, medical, or intensive care unit); laboratory and microbiological findings; treatment; and outcomes (mortality). Data were collected from patient admission until discharge due to improvements or death.

Multidrug resistance includes strains resistant to 3 or more classes of antibiotics, particularly carbapenems (14–16).

### Outcomes

The primary outcome of the analysis was BSI all-cause mortality at ≤ 30 days. All-cause mortality at ≤ 30 days was estimated, with the first day defined as the date of collection of the positive blood culture. The primary secondary outcomes included the administration of an ineffective empirical treatment (empirical treatment failure), development of resistance, and length of hospital stay.

Empirical treatment consists of administering an antibiotic before the blood culture results and susceptibility pattern are known, given the patient's severe clinical condition.

To classify a patient as having received ineffective empirical treatment (treatment failure), at least one of the following criteria was considered:

(a) Susceptibility Pattern

The isolated bacterium was resistant to the empirically administered antibiotic.

(b) Antibiotic administration

The antibiotic was not administered within the first 24 h of suspected bacterial infection (BSI) (immediately after the blood culture was taken).

(c) Treatment duration (number of consecutive days of therapy)

Although the isolated bacteria were susceptible to the prescribed antibiotic, the patient did not receive treatment for seven consecutive days.

(d) Infection source

The catheter or source of infection should have been removed.

Inclusion criteria: Patients with clinically and microbiologically confirmed BSI, isolated from a GNB. With positive monomicrobial blood cultures, one was obtained from the CVC and another from peripheral venipuncture. With age 18 years or older, regardless of sex, with or without comorbidities, with one or more hospitalization conditions upon admission, with a hospital stay in any medical or surgical department, including the hospital's five intensive care units (general ICU, burn ICU, transplant ICU, coronary ICU, and post-surgical heart ICU), as well as with special conditions during hospitalization, such as the use of invasive mechanical ventilation or parenteral nutrition.

Exclusion criteria: Patients with clinically and microbiologically ruled-out BSI; patients with central venous catheter colonization, with blood cultures showing polymicrobial isolation, or with isolation of a microorganism other than GNB. The patient was on antibiotic treatment (48 h before sample collection)—patients with incomplete data or patients who have requested voluntary discharge within the established retrospective follow-up period.

### Blood culture

The peripheral venipuncture site and CVC are disinfected with chlorhexidine or 2% iodine tincture. A 20 to 30 ml blood sample is drawn, which is required for a set of two vials per collection (CVC and peripheral venipuncture): one for aerobic microorganisms and one for anaerobic microorganisms. Blood culture kits are transported to the laboratory at room temperature as soon as possible. Microbiological isolation and antimicrobial susceptibility were obtained by the hospital laboratory using the microdilution method (Vitek 2, BioMérieux). To classify antibiotic susceptibility/resistance, the 30th edition (2020) of the Clinical and Laboratory Standards Institute was used (14–16).

For a BSI infection to be considered associated with a CVC, the blood culture from the CVC must be positive at least 120 min before the blood culture from the peripheral venipuncture, provided that the bacteria are the same. If the bacteria grow earlier during peripheral venipuncture, it is considered a secondary BSI (due to another nosocomial infection with the same bacteria isolated) (3, 8, 17).

An adaptation of the classification proposed by Falcone et al. was made for GNB in the following categories: (1) Carbapenem-susceptible Gram-negative bacilli (CS-GNB); (2) Carbapenem-resistant *P. aeruginosa* (CRPA) (due to carbapenemases and not porins, according to the results of the antibiogram); (3) Carbapenem-resistant *A. baumannii* (CRAB); (4) Carbapenem-resistant *Enterobacterales* (CRE); and (5) Other Fermenting GNB (*Stenotrophomonas maltophilia* and *Alcaligenes faecalis*) (18).

Carbapenem- resistance cut-off values by group: CRPA, carbapenems (imipenem and meropenem) ≥8 μg/ml Minimum Inhibitory Concentration (MIC); CRE, carbapenems (imipenem and meropenem) ≥4 μg/ml, and for ertapenem ≥2 μg/ml MIC; and CRAB, carbapenems (imipenem and meropenem) ≥8 μg/ml MIC. Resistance cut-off values for polymyxins (MIC), ≥4 μg/ml for the most critical members of *Enterobacterales* and *Acinetobacter* spp., and for *P. aeruginosa*, were considered (15).

Regarding *P. aeruginosa*, porin-mediated carbapenem resistance was inferred phenotypically based on the antibiogram pattern, with susceptibility to ceftazidime, cefepime, or piperacillin-tazobactam (19).

A description and analysis of two BSI outbreaks due to the isolation of an opportunistic GNB, which were observed during the cohort follow-up, were included.

### Statistical analysis

Qualitative variables were analyzed using the χ^2^ test or Fisher's exact test. Quantitative variables with normal distributions were summarized using the arithmetic mean and standard deviation and compared using the Student's *t*-test. Quantitative variables with non-normal distributions were summarized using medians and interquartile ranges, and comparisons between groups were performed using the Mann-Whitney U test. Exact 95% confidence intervals (CIs) were used to compare groups. A bilateral *p*-value < 0.05 was considered statistically significant. To establish cut-off points for converting quantitative variables to qualitative variables, ROC (receiver operating characteristic) curves were used.

The following statistical analyses were performed using univariate models:

(1) Analysis of the risk factors associated with mortality among survivors and non-survivors due to BSI who died within ≤ 30 days, with the first day defined as the date of collection of the positive blood culture. (2) Analysis of the risk factors for presenting BSI due to CR-GNB vs. CS-GNB (the isolates of *S. maltophilia* were excluded because it has intrinsic resistance to carbapenems; *A. faecalis* was also excluded). (3) Analysis of the poor prognostic factors in patients with BSI due to MDR-GNB, stratified according to the bacilli isolated. (4) And, an additional analysis to review the conditions of patients at the Transplant Unit with BSI due to *Achromobacter xylosoxidans*.

Statistically significant risk factors associated with mortality in univariate analysis (among survivors and non-survivors) were analyzed using survival curves (Cox regression) and the adjusted Cox Proportional Hazard Model (SPSS 25, IBM, Armonk, NY, USA).

## Results

**Figure 1:**
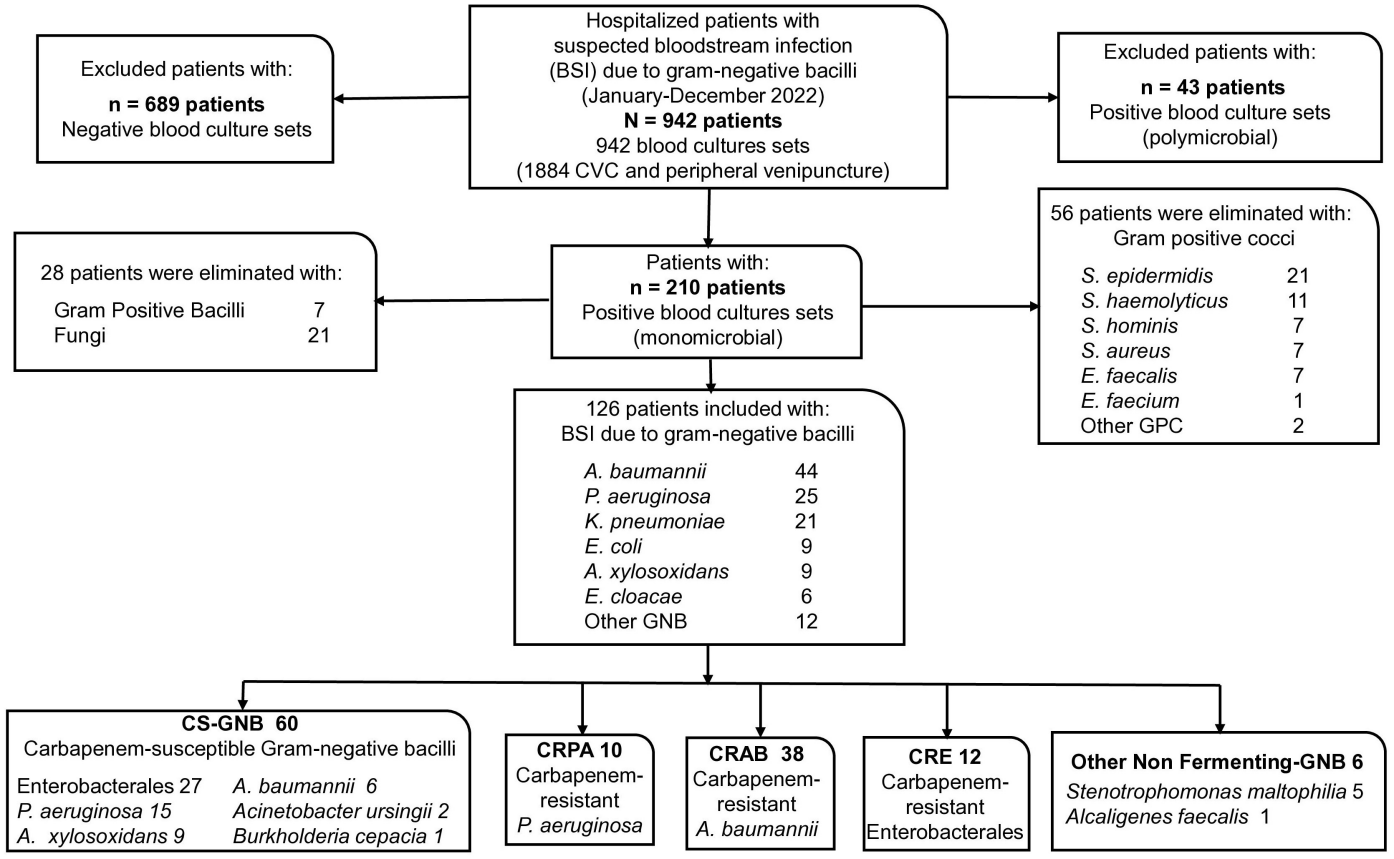
Participant flowchart. CVC, Central venous catheter.

The flowchart (Figure 1) presents the results of the review of the physical and electronic records of the included patients, along with the microbiological findings. A total of 942 hospitalized patients with suspected bacteremia were identified from 1884 cultures performed during the study period (942 blood cultures). Two hundred ten patients had positive monomicrobial blood cultures, meeting the clinical and laboratory criteria for bacteremia. Fifty-six patients with Gram-positive cocci, 7 with Gram-positive bacilli, and 21 with fungi were excluded. The remaining 126 patients with BSI and GNB isolation were included.

In the 126 blood cultures included, the Gram-negative bacteria (GNB) found, ordered by frequency, were 44 isolates of *A. baumannii* (34.9%), 25 of *P. aeruginosa* (19.8%), 21 of *K. pneumoniae* (16.7%), 9 of *E.coli* (7.1%), 9 of *A. xylosoxidans* (7.1%), 6 of *Enterobacter cloacae* (4.8%), and 12 of other GNB (9.5%).

According to classification, 60 isolates (47.6%) were classified as CS-GNB; 27 belonged to the order *Enterobacterales*: 15 of *P. aeruginosa*, 9 of *A. xylosoxidans*, 6 of *A. baumannii*, 2 of *A. ursingii*, and 1 of *B. cepacia*. And 66 isolates (52.4%) were found to be CR-GNB: 10 CRPA isolates, 38 CRAB isolates, 12 CRE isolates, and six other non-fermenting GNB isolates (5 *S. maltophilia* and 1 *A. faecalis*) (Figure 1).

Table 1 presents the demographic characteristics, comorbidities, complications, laboratory findings, and outcomes at ≤ 30 days of follow-up in patients with BSI, along with blood culture isolates and antimicrobial resistance. Of the patients included, 67 (53.1%) were male and 59 (46.9%) female, with an age range of 18 to 82 years and a mean age of 46.5 ± 15.7 years.

**Table 1:** Demographic characteristics, comorbidities, complications, laboratory findings, and mortality at ≤ 30 days of follow-up for patients with bloodstream infection; blood culture isolates and antimicrobial resistance.

**Variables**	**Total *N* = 126 (%)**	**Survivors *N* = 90 (%)**	**Non-survivors *N* = 36 (28.6)**	**OR (95% CI)**	***p***
Man	67 (53.2)	48 (53.3)	19 (52.8)		0.555
Age (years), mean ± SD	46.5 ± 15.7	44.4 ± 16.0	51.8 ± 13.8		0.012^a^
Age ≥46 years	61 (48.4)	35 (38.9)	26 (72.2)	4.1 (1.8–9.5)	0.001
**Comorbidities and conditions at admission**					
Immunocompromise	86 (68.3)	62 (68.9)	24 (66.7)		0.483
Arterial hypertension	59 (46.8)	43 (47.8)	16 (44.4)		0.445
Diabetes mellitus	35 (27.8)	18 (20.0)	17 (47.2)	3.6 (1.6–8.2)	0.003
Vascular venous exhaustion	31 (24.6)	22 (24.4)	9 (25.0)		0.558
Chronic kidney disease	28 (22.2)	24 (26.7)	4 (11.1)		0.062
Hemodialysis	27 (21.4)	21 (23.3)	6 (16.7)		0.285
SAH/TBI/brain hemorrhage	23 (18.3)	16 (17.8)	7 (19.4)		0.505
Patients admitted for burns	21 (16.7)	16 (17.8)	5 (13.9)		0.792
Kidney transplant	18 (14.3)	18 (20.0)	0		NS
Neoplasia/leukemia	9 (7.1)	6 (6.7)	3 (8.3)		0.714
Short bowel syndrome	9 (7.1)	8 (8.9)	1 (2.8)		0.444
Ischemic heart disease	8 (6.3)	4 (4.4)	4 (11.1)		0.224
**Conditions during hospitalization**					
Surgery hospitalization ward	87 (69.0)	60 (66.7)	27 (75.0)		0.244
Septic shock	86 (68.3)	51 (56.7)	35 (97.2)	26.8 (3.5–204)	0.000
Invasive mechanical ventilation	76 (60.3)	46 (51.1)	30 (83.3)	4.8 (1.8–12.6)	0.001
Medical hospitalization ward	38 (30.2)	29 (32.2)	9 (25.0)		0.283
Intensive care unit	56 (44.4)	37 (41.1)	19 (52.8)		0.161
Parenteral nutrition	18 (14.3)	9 (10.0)	9 (25.0)	3.0 (1.1–8.3)	0.033
Post-cardiac arrest syndrome	9 (7.1)	4 (4.4)	5 (13.9)		0.117
Central venous catheter (d), median (IQR)	20 (12–34)	19 (13–34)	23 (11–35)		0.846
Hospitalization (d), median (IQR)	31 (20–51)	36 (23–53)	25 (14–44)		0.055^b^
Hospital admission to BSI (d), median (IQR)	13 (7–28)	13 (6–25)	15 (9–34)		0.197^b^
BSI to mortality ≤ 30-d, median (IQR)	14 (7–23)	16 (10–30)	6 (2–18)		0.000^b^
**Laboratory findings**					
Blood leukocytes (cells/μl), mean ± SD	15.9 ± 7.6	16.2 ± 7.4	15.2 ± 8.2		0.595^a^
Procalcitonin (ng/ml), median (IQR)	5.7 (1.9–18.5)	4.2 (1.7–18.7)	7.3 (3.3–19.3)		0.371^b^
**Resistance by type of bacteria**					
*A. baumannii* (CRAB and non-CRAB)	44 (34.9)	25 (27.8)	19 (52.8)	2.9 (1.3–6.5)	0.008
*P. aeruginosa* (CRPA and non-CRPA)	25 (19.8)	22 (24.4)	3 (8.3)		NS
*Enterobacterales* (CRE and non-CRE)	39 (31.0)	24 (26.7)	15 (41.7)		0.077
Carbapenem-resistant *P. aeruginosa* (CRPA)	10 (7.9)	8 (8.9)	2 (5.6)		0.723
Carbapenem-resistant *A. baumannii* (CRAB)	38 (30.2)	19 (21.1)	19 (52.8)	4.2 (1.8–9.6)	0.001
Carbapenem-resistant *Enterobacterales* (CRE)	12 (9.5)	5 (5.6)	7 (19.4)	4.1 (1.2–14.0)	0.038
Other NGF-GNB	6 (4.8)	2 (2.2)	4 (11.1)		0.055
CR-GNB	66 (52.4)	34 (37.8)	32 (88.9)	13.2 (4.3–40.5)	0.000
Carbapenem-resistant CRAB and CRE	50 (39.7)	24 (26.7)	26 (72.2)	7.2 (3.0–17.0)	0.000
**Antibiotic resistance**					
Meropenem resistance	67/121 (55.4)	38/89 (42.7)	29/32 (90.6)	13.0 (3.7–45.8)	0.000
Piperacillin/tazobactam resistance	65/113 (57.5)	36/82 (43.9)	29/31 (93.5)	18.5 (4.1–82.9)	0.000
Resistance to cefepime	76/119 (63.9)	46/87 (52.9)	30/32 (93.8)	13.4 (3.0–59.4)	0.000
Resistance to cefoxitin	45/76 (59.2)	29/57 (50.9)	16/19 (84.2)	5.1 (1.4–19.6)	0.014
Resistance to 3rd-generation cephalosporins	98/118 (83.1)	66/86 (76.7)	32/32 (100.0)		NS
Resistance to quinolones	83/123 (67.5)	53/87 (60.9)	30/36 (83.3)	3.2 (1.2–8.5)	0.012
Resistance to aminoglycosides	59/118 (50.0)	38/86 (44.2)	21/32 (65.6)	2.4 (1.0–5.6)	0.031
Tigecycline resistance	29/87 (33.3)	23/61 (37.7)	6/26 (23.1)		0.140
Colistin resistance	3/89 (3.4)	2/62 (3.2)	1/27 (3.7)		1.000
**Outcomes**					
Ineffective empirical treatment	48/126 (38.1)	15/90 (16.7)	33/36 (91.7)	55.0 (15.0–203)	0.000
From bloodstream infection to mortality ≤ 30-d	36/126 (28.6)	0	36/36 (100.0)		NA
Overall mortality of patients with BSI	46/126 (36.5)	10/90 (11.1)	36/36 (100.0)		NA

SAH, Subarachnoid hemorrhage; TBI, Traumatic brain injury; BSI, Bloodstream infections; CVC, Central venous catheter; d, days; CS-GNB, Carbapenem-susceptible Gram-negative bacilli; CR-GNB, Carbapenem-resistant Gram-negative bacilli; Other NGF-GNB, Other non-fermenting Gram-negative bacilli; ng/ml, nanograms/milliliter; OR, Odds ratio; CI, Confidence interval; SD, Standard deviation; IQR, Interquartile range; NS, Not significant; NA, Not applicable.

^a^Student's *t* statistical test.

^b^Mann–Whitney *U* test.

The most common comorbidities listed among the patients in the cohort, in descending order, were 86 patients (68.3%) who were immunocompromised, 59 (46.8%) with arterial hypertension, 35 (27.8%) with diabetes mellitus, and 31 (24.6%) with vascular access exhaustion. Eighty-seven patients (69.0%) came from surgical hospitalization areas, and 38 patients (30.2%) came from medical hospitalization areas; 86 (68.3%) had septic shock, 76 (60.3%) required invasive mechanical ventilation support, 56 (44.4%) were admitted to an intensive care unit, 18 (14.3%) received parenteral nutrition, and 9 (7.1%) had a post-cardiac arrest syndrome.

The median hospitalization for the selected patients was 31 days (IQR 20–51). No deaths were observed among patients with BSI admitted to the Renal Transplant Intensive Care Unit. A median of 6 days from BSI to mortality ≤ 30 days was estimated (IQR 2–18 d, *p* = 0.000) (Mann–Whitney *U* test). A mortality rate of 28.6% (36/126) from all causes was found at ≤ 30 days; of these deaths, 32/36 (88.9%) were CR-GNB (Table 1).

In the univariate analysis between patients with bacteremia who did not survive and those who did survive, it was found that age ≥46 years (OR 4.1 CI 1.8–9.5, *p* = 0.001), diabetes mellitus (OR 3.6 CI 1.6–8.2, *p* = 0.003), septic shock (OR 26.8 CI 3.5–204, *p* = 0.000), use of invasive mechanical ventilation (OR 4.8 CI 1.8–12.6, *p* = 0.001), need for parenteral nutrition (OR 3.0 CI 1.1–8.3, *p* = 0.033), presence of CR-GNB (OR 13.2 CI 4.3–40.5, *p* = 0.000), and having received ineffective empirical treatment (OR 55.0 CI 15.0–203, *p* = 0.000) were statistically significant factors for mortality risk. Among patients who did not survive ≤ 30 days, 19/36 (52.8%) had CRAB (OR 4.2, CI 1.8–9.6, *p* = 0.001) (Table 1).

**Figure 2:**
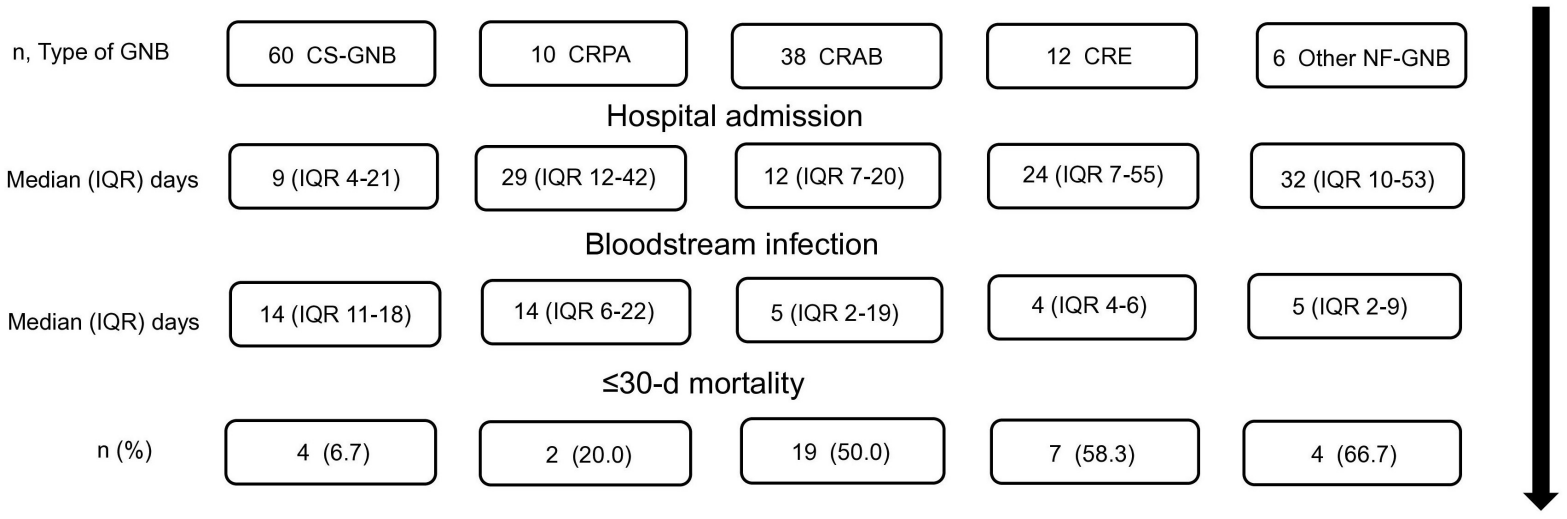
Timeline of the study, patient's hospital admission, follow-up, and outcomes. CS-GNB, Carbapenem-susceptible Gram-negative bacilli; CRPA, carbapenem-resistant *P. aeruginosa*; CRAB, Carbapenem-resistant *A. baumannii*; CRE, carbapenem-resistant *Enterobacterales*; Other NF-GNB, Other Non-fermenting Gram-negative bacilli; IQR, Interquartile range; d, days.

Figure 2 shows a timeline of the study, including hospital admission, follow-up, and patient outcomes. According to the proposed classification for the groups of isolated bacilli, the following mortality outcomes were observed: In the CS-GNB group 4/60 patients (6.7%), in the CRPA group 2/10 patients (20.0%), in the CRAB group 19/38 patients (50.0%), in the CRE group 7/12 (58.3%), and in the other NF-GNB group 4/6 (66.7%) who died within ≤ 30 days.

**Figure 3:**
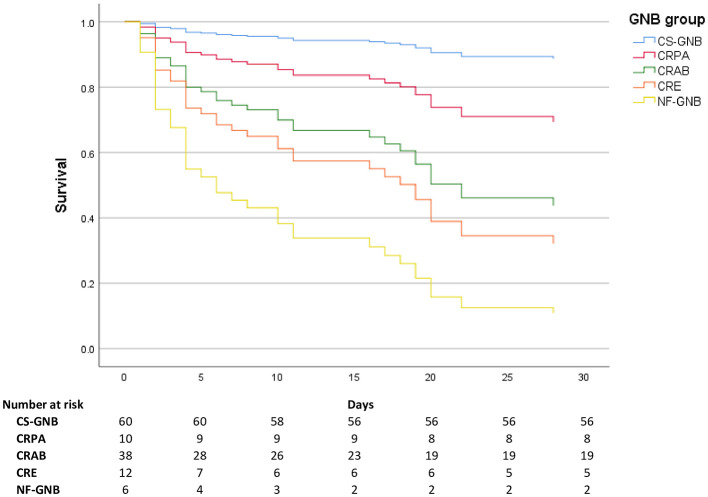
Survival curves by bacterial group. CS-GNB, Carbapenem-susceptible Gram-negative bacilli; CRPA, carbapenem-resistant *P. aeruginosa*; CRAB, Carbapenem-resistant *A. baumannii*; CRE, carbapenem-resistant *Enterobacterales*; Other NF-GNB, Other Non-fermenting Gram-negative bacilli.

Survival curves for all-cause mortality at ≤ 30 days among patients with BSI, stratified by GNB group, are presented in Figure 3.

**Figure 4:**
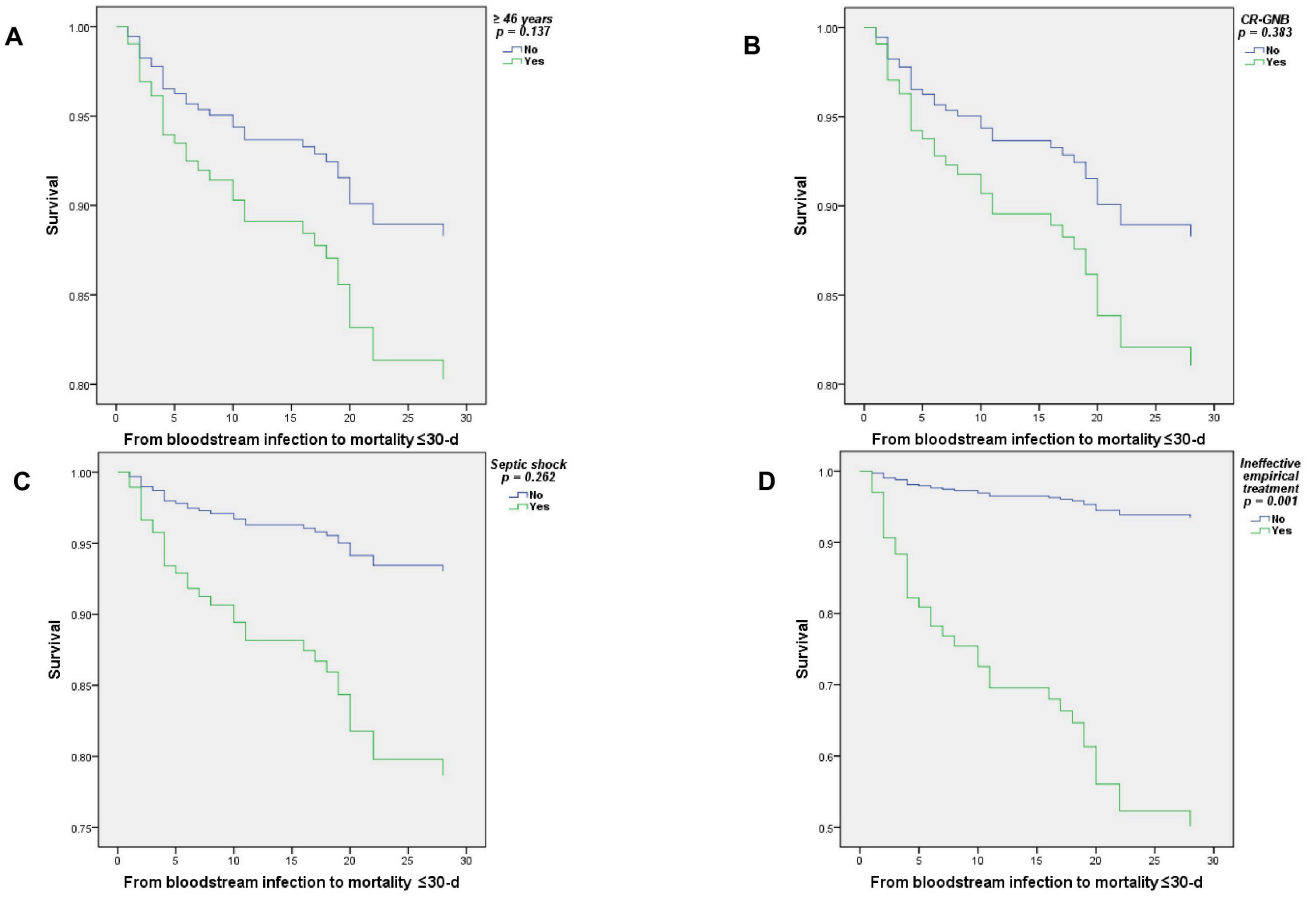
Cox regression survival curves of patients with BSI due to multidrug-resistant Gram-negative bacilli, ≤ 30-d mortality. **(A)** ≥46 years, **(B)** CR-GNB, **(C)** Septic shock, and **(D)** Ineffective empirical treatment.

Figure 4 displays the survival curves (Cox regression) for the principal risk factors associated with all-cause mortality at ≤ 30 days in patients with BSI due to MDR-GNB: age ≥46 years, CR-GNB infection, ineffective empirical treatment, and septic shock. Where having received an ineffective empirical treatment was statistically significant (*p* = 0.001).

Table 2 shows the characteristics of patients with BSI due to CS-GNB vs. CR-GNB isolates.

**Table 2:** Demographic characteristics, comorbidities, complications, and laboratory findings of patients with bloodstream infections due to carbapenem-susceptible Gram-negative bacilli vs. carbapenem-resistant Gram-negative bacilli.

**Variables**	**Total *n* = 120 (%)**	**CS-GNB *n* = 60 (50)**	**CR-GNB *n* = 60 (50)**	**OR (95% CI)**	***p***
Man	65 (54.2)	35 (58.3)	30 (50.0)		0.232
Age (years), mean ± SD	47 ± 16	46 ± 17	48 ± 15		0.483^a^
Age ≥46 years	59 (49.2)	27 (45.0)	32 (53.3)		0.233
**Comorbidities and conditions at admission**					
Immunocompromise	81(67.5)	42 (70.0)	39 (65.0)		0.348
Arterial hypertension	58 (48.3)	33 (55.0)	25 (41.7)		0.100
Diabetes mellitus	32 (26.7)	12 (20.0)	20 (33.3)		0.074
Vascular access exhaustion	29 (24.2)	13 (21.7)	16 (26.7)		0.335
Chronic kidney disease	27 (22.5)	19 (31.7)	8 (13.3)		NS
Hemodialysis	26 (21.7)	19 (31.7)	7 (11.7)		NS
SAH/TBI/brain hemorrhage	22 (18.3)	6 (10.0)	16 (26.7)	3.3 (1.2–9.1)	0.016
Patients admitted for burns	20 (16.7)	9 (15.0)	11 (18.3)		0.404
Kidney transplant	18 (15.0)	16 (26.7)	2 (3.3)		NS
Neoplasia/leukemia	9 (7.5)	3 (5.0)	6 (10.0)		0.491
Short bowel syndrome	9 (7.5)	5 (8.3)	4 (6.7)		1.000
Ischemic heart disease	8 (6.7)	3 (5.0)	5 (8.3)		0.717
**Conditions during hospitalization**					
Central venous catheter (d), median (IQR)	19 (12–35)	20 (12–32)	19 (11–38)		0.973^b^
Hospitalization in the surgical area	82 (68.3)	34 (56.7)	48 (80.0)	3.1 (1.4–6.9)	0.005
Septic shock	82 (68.3)	32 (53.3)	50 (83.3)	4.4 (1.9–10.2)	0.000
Invasive mechanical ventilation	73 (60.8)	24 (40.0)	49 (81.7)	6.7 (3.0–15.4)	0.000
Hospitalization in the medical area	37 (30.8)	25 (41.7)	12 (20.0)		NS
Intensive care unit	52 (43.3)	18 (30.0)	34 (56.7)	3.1 (1.4–6.5)	0.003
Parenteral nutrition	17 (14.2)	7 (11.7)	10 (16.7)		0.301
Ventilator-associated pneumonia	35 (29.2)	12 (20.0)	23 (38.3)	2.5 (1.1–5.6)	0.022
Skin and soft tissue infection	18 (15.0)	8 (13.3)	10 (16.7)		0.399
Post-cardiac arrest syndrome	9 (7.5)	4 (6.7)	5 (8.3)		1.000
Hospitalization (d), mean ± SD	37 ± 22	33 ± 19	40 ± 24		0.095^a^
Hospital admission to BSI (d), median (IQR)	13 (6–25)	9 (4–21)	14 (8–29)		0.048^b^
BSI to mortality ≤ 30–d, median (IQR)	7 (14–25)	14 (10–23)	15 (4–30)		0.987^b^
**Antibiotic resistance**					
Meropenem resistance	66/118 (55.9)	6/58 (10.3)	60 (100.0)		NS
Resistance to 3rd generation cephalosporins	97/117 (82.9)	38/57 (66.7)	59 (98.3)	29.5 (3.8–229)	0.000
Resistance to quinolones	82/117 (70.1)	25/58 (43.1)	57/59 (96.6)	37.6 (8.4–169)	0.000
Resistance to aminoglycosides	58/117 (49.6)	20/58 (34.5)	38/59 (64.4)	3.4 (1.6–7.4)	0.001
Tigecycline resistance	26/86 (33.7)	10/41 (24.4)	19/45 (42.2)		0.064
Colistin resistance	3/89 (3.4)	1/38 (2.6)	2/51 (3.9)		1.000
**Laboratory findings**					
Blood leukocytes × 10^3^ cells/μl, mean ± SD	15.8 ± 7.5	16.8 ± 7.9	14.9 ± 7.0		0.155^a^
Procalcitonin (ng/ml), median (IQR)	5.5 (1.9–17.7)	3.4 (1.5–18.0)	6.6 (2.2–17.7)		0.452^b^
Ineffective empirical treatment	45/120 (37.5)	4/60 (6.7)	41/60 (68.3)	30.2 (9.6–92.5)	0.000
From bloodstream infection to mortality ≤ 30-d	32 (26.7)	4 (6.7)	28 (46.7)	12.3 (3.9–38.1)	0.000
Overall mortality of patients with BSI	42 (35.0)	8 (13.3)	34 (56.7)	8.5 (3.4–21.0)	0.000

SAH, Subarachnoid hemorrhage; TBI, Traumatic brain injury; BSI, bloodstream infections; CVC, Central venous catheter; d, days; CS-GNB, Carbapenem-susceptible Gram-negative bacilli; CR-GNB, Carbapenem-resistant Gram-negative bacilli; ng/ml, nanograms/milliliter; OR, Odds ratio; CI, Confidence interval; SD, Standard deviation; IQR, Interquartile range; NS, Not significant.

^a^Student's *t* statistical test.

^b^Mann–Whitney *U* test.

The following risk factors (according to univariate analysis) were identified as statistically significant for patients with CR-GNB: subarachnoid hemorrhage (SAH), head trauma (TBI), cerebral hemorrhage (OR 3.3; CI 1.2–9.1; p = 0.016), conditions during hospitalization, particularly in surgical areas (OR 3.1; CI 1.4–6.9; *p* = 0.005), septic shock (OR 4.4; CI 1.9–10.2; *p* = 0.000), invasive mechanical ventilation (IMV) (OR 6.7; CI 3.0–15.4; *p* = 0.000) and intensive care unit (general and burn) (OR 3.1; CI 1.4–6.5; *p* = 0.003). Patients with ventilator-associated pneumonia (OR 2.5; CI 1.1–5.6; *p* = 0.022). The number of days from admission to the day of a positive blood culture was also statistically significant (*p* = 0.048). We found that having received ineffective empirical treatment was statistically significant (OR 30.2, CI 9.6–92.5, *p* = 0.000), as well as mortality ≤ 30 days by BSI (OR 12.3, CI 3.9–38.1, *p* = 0.000) and overall mortality (OR 8.5, CI 3.4–21.0, *p* = 0.000).

Table 3 shows the poor prognostic factors for death in patients with BSI according to the four main categories specified. For patients who had a CRAB isolation, 33/38 (86.8%) had septic shock, and 13/38 (34.2%) had SAH/TBI/brain hemorrhage. Statistical significance was observed in those who presented SAH/TBI/brain hemorrhage (OR 4.1, IC 1.6–10.4, *p* = 0.003), IMV (OR 6.9, IC 2.5–19.3, *p* = 0.000), septic shock (OR 4.4, IC 1.6–12.2, *p* = 0.003), and intensive care unit (OR 2.6, IC 1.2–5.6, *p* = 0.014). One hundred percent of CRAB were resistant to piperacillin/tazobactam, cefepime, third-generation cephalosporins, and quinolones; 65.8% were resistant to aminoglycosides, and 44.7% were resistant to tigecycline, with statistical significance for resistance to aminoglycosides (OR 2.6, CI 1.2–5.8, *p* = 0.015), having received an ineffective empirical treatment (OR 5.4, CI 2.4–12.4, *p* = 0.000), and not surviving the first 30 days of BSI (OR 4.2, CI 1.8–9.6, *p* = 0.001).

**Table 3:** Poor prognostic factors in patients with bloodstream Infection due to multidrug-resistant Gram-negative bacilli.

**Variables**	**Carbapenem-resistant** ***A. baumannii*** **(CRAB)**			**Carbapenem-resistant** ***Enterobacterales*** **(CRE)**		
	***n*** = **38 (%)**	**OR (95% CI)**	***p***	***n*** = **12 (%)**	**OR (95% CI)**	***p***
Diabetes mellitus	11 (28.9)		0.504	6 (50.0)		0.075
SAH/TBI/brain hemorrhage	13 (34.2)	4.1 (1.6–10.4)	0.003	3 (25.0)		0.458
Chronic kidney disease	4 (10.5)		0.060	3 (25.0)		0.728
Hemodialysis	4 (10.5)		0.060	2 (16.7)		1.000
Kidney transplant	1 (2.6)		NS	0		NS
Invasive mechanical ventilation	33 (86.8)	6.9 (2.5–19.3)	0.000	8 (66.7)		0.762
Septic shock	33 (86.8)	4.4 (1.6–12.2)	0.003	9 (75.0)		0.751
Intensive care unit	23 (60.5)	2.6 (1.2–5.6)	0.014	7 (58.3)		0.368
Piperacillin/tazobactam resistance	38 (100.0)		NS	11/11 (100.0)		NS
Resistance to cefepime	38 (100.0)		NS	11 (91.7)		0.054
Resistance to 3rd-generation cephalosporins	38 (100.0)		NS	12 (100.0)		NS
Resistance to quinolones	38 (100.0)		NS	11/11 (100.0)		NS
Resistance to aminoglycosides	25 (65.8)	2.6 (1.2–5.8)	0.015	5/11 (45.5)		1.000
Tigecycline resistance	17 (44.7)		0.040	2/7 (28.6)		1.000
Colistin resistance	0		NS	1/9 (11.1)		0.277
Ineffective empirical treatment	25 (65.8)	5.4 (2.4–12.4)	0.000	9 (75.0)	5.8 (1.5–22.5)	0.010
From bloodstream infection to mortality ≤ 30-d	19 (50.0)	4.2 (1.8–9.6)	0.001	7 (58.3)	4.1 (1.2–14.0)	0.038
**Variable**	**Carbapenem-susceptible Gram-negative bacilli (CS-GNB)**			**Carbapenem-resistant** ***P. aeruginosa*** **(CRPA)**		
	***n*** = **60 (%)**	**OR (95% CI)**	***p***	***n*** = **10 (%)**	**OR (95% CI)**	***p***
Diabetes mellitus	12 (20.0)		NS	3 (30.0)		1.000
SAH/TBI/brain hemorrhage	6 (10.0)		NS	0		NS
Chronic kidney disease	19 (31.7)		NS	1 (10.0)		0.456
Hemodialysis	19 (31.7)		NS	1 (10.0)		0.688
Kidney transplant	16 (26.7)		NS	1 (10.0)		1.000
Invasive mechanical ventilation	24 (40.0)		NS	8 (80.0)		0.313
Septic shock	32 (53.3)		NS	8 (80.0)		0.501
Intensive care unit	18 (30.0)		NS	4 (40.0)		1.000
Piperacillin/tazobactam resistance	9/56 (16.1)		NS	6/7 (85.7)		0.235
Resistance to cefepime	17/58 (29.3)		NS	9 (90.0)		0.092
Resistance to 3rd-generation cephalosporins	38/57 (66.7)		NS	9 (90.0)		1.000
Resistance to quinolones	25/58 (43.1)		NS	8 (80.0)		0.721
Resistance to aminoglycosides	20/58 (34.5)		NS	8 (80.0)		0.094
Tigecycline resistance	10/41 (24.4)		0.074	NA		NA
Colistin resistance	1/38 (2.6)		1.000	1/8 (12.5)		0.249
Ineffective empirical treatment	4 (6.7)		NS	7 (70.0)		NS
From bloodstream infection to mortality ≤ 30-d	4 (6.7)		NS	2 (20.0)		0.723

SAH, Subarachnoid hemorrhage; TBI, Traumatic brain injury; BSI, bloodstream infections; d, days; OR, Odds ratio; CI, Confidence interval; SD, Standard deviation; IQR, Interquartile range; NS, Not significant; NA, Not applicable.

For patients with CRE isolation, having received an ineffective empirical treatment (OR 5.8, IC 1.5–22.5, *p* = 0.010) and death within ≤ 30 days of BSI (OR 4.1, IC 1.2–14.0, *p* = 0.038) were estimated to be statistically significant. No poor prognostic factors were observed in patients with BSI in the CS-GNB or CRPA groups.

To run the adjusted Cox proportional hazards model, the significant variables from the previous univariate models were included: age ≥46 years, diabetes mellitus, septic shock, use of invasive mechanical ventilation, BSI due to CR-GNB, receipt of parenteral nutrition, and prescription of ineffective empirical treatment. The final model included the variables: age ≥46 years, septic shock, BSI due to CR-GNB, and ineffective empirical treatment. Furthermore, for all variables, 95% confidence intervals and *p*-values ≤ 0.05 were considered statistically significant.

Table 4 summarizes the results of this analysis, finding that having received ineffective empirical treatment was associated with a statistically significant increase in mortality (HR = 10.2, 95% CI = 2.6–39.9, *p* = 0.001).

**Table 4:** Mortality risks according to the adjusted Cox proportional hazards model.

**Variable**	**HR (95% CI)**	***p***
≥46 years	1.8 (0.84–3.7)	0.137
Septic shock	3.3 (0.41–27.2)	0.262
CR-GNB	1.7 (0.52–5.4)	0.383
Ineffective empirical treatment	10.2 (2.6–39.9)	0.001

CI, Confidence interval; CR-GNB, Carbapenem-resistant Gram-negative bacilli; HR, Hazard Ratio, adjusted for age ≥46 years, diabetes mellitus, septic shock, invasive mechanical ventilation; CR-GNB, parenteral nutrition, and ineffective empirical treatment.

Two outbreaks involving a total of 9 patients were detected in the Intensive Care Unit for kidney transplant patients: March 2022 (6 cases) and September 2022 (3 cases), both involving CVC-associated BSI due to *A. xylosoxidans*. Table 5 shows the demographic data, comorbidities, and conditions of these patients. Statistically significant resistance to tigecycline was found for *A. xylosoxidans* (OR 8.9, IC 1.7–46.3, *p* = 0.006). No death events were recorded due to this GNB.

**Table 5:** Patients with central venous catheter-associated bacteremia due to *Achromobacter xylosoxidans*.

**Variable**	***n* = 9**	**OR (95 % CI)**	***p***
Age, mean ± SD	35 ± 8.4		0.027^a^
Diabetes mellitus	1 (11.1)		0.443
SAH/TBI/brain hemorrhage	0		NA
Chronic kidney disease	9 (100.0)		NS
Hemodialysis	9 (100.0)		NS
Kidney transplant	9 (100.0)		NS
**Conditions during hospitalization**			
Invasive mechanical ventilation	0		NA
Septic shock	3 (33.3)		NS
Transplant Intensive Care Unit	9 (100.0)		NS
BSI associated with the central venous catheter	9 (100.0)		NS
Endocarditis	0		NA
Hospitalization (d), mean ± SD	15 ± 5		0.000^a^
Hospital admission to BSI (d), median (IQR)	4 (3–7)		0.001^b^
BSI to discharge or mortality ≤ 30-d, mean ± SD	9 ± 5.4		0.071^a^
Central venous catheter (d), mean, ± SD	13 ± 3.6		0.005^a^
**Laboratory findings**			
Blood leukocytes (cells/μl), mean ± SD	16.6 ± 9.9		0.822
Procalcitonin (ng/ml), median (IQR)	2.5 (1.1–18.9)		0.315
**Antibiotic resistance**			
Resistance to carbapenems	0		NA
Piperacillin/tazobactam resistance	0		NA
Resistance to cefoxitin	2/6 (33.3)		0.218
Resistance to cefepime	1 (11.1)		NS
Resistance to 3rd-generation cephalosporins	7/8 (87.5)		1.000
Resistance to quinolones	9 (100.0)		NS
Resistance to aminoglycosides	9 (100.0)		NS
Tigecycline resistance	7 (77.8)	8.9 (1.7–46.3)	0.006
Colistin resistance	0		NA
**Outcomes**			
Ineffective empirical treatment	0		NA
From bloodstream infection to mortality ≤ 30-d	0		NA

Outbreaks in March and September 2022.

SAH, Subarachnoid hemorrhage; TBI, Traumatic brain injury; BSI, Bloodstream infections; ng/ml, nanograms/milliliter; SD, Standard deviation; CI, Confidence interval; IQR, Interquartile range; d, days; NS, Not significant; NA, Not applicable; OR, Odds ratio; CI, Confidence interval; SD, Standard deviation; IQR, Interquartile range; NS, Not significant; NA, Not applicable.

^a^Student's *t* statistical test.

^b^Mann–Whitney *U* test.

## Discussion

Of 210 patients with positive monomicrobial blood cultures who met the clinical and microbiological criteria for BSI, 40% (84/210) were excluded, with positive isolates of Gram-positive cocci, Gram-positive bacilli, and fungi. One hundred and twenty-six patients with BSI due to GNB were selected, representing 60% (126/210) of the total.

Our hospital provides tertiary care and serves as a referral center. We routinely receive patients with complex, advanced-stage diseases. We found a mortality of 28.6% in ≤ 30 days due to the GNB BSI. The estimated mortality is higher than that reported in other published studies, which reported 21.6% in ≤ 30 days for GNB-BSI in a multicenter study in Italian hospitals (12) and 17.4% in ≤ 30 days in a cohort study in Spanish hospitals (18).

Mortality due to BSI was related to a high frequency of patients with infection by CR-GNB, mainly CRAB; according to the results of the univariate analysis, mortality was 13.2 (CI 4.3–40.5, *p* = 0.000) times higher in patients with CR-GNB isolation and 4.2 (CI 1.8–9.6, *p* = 0.001) times higher in those with CRAB.

Male gender and advanced age have been reported as a frequent demographic profile of patients with BSI. We also found slightly more men but a younger patient group, with statistical significance only in univariate analysis. The younger age found in our patients is notable, which we consider has to do with the severe conditions with which patients are referred to our hospital, since it is documented that older adults have a higher risk of CR-GNB infection, given that they usually have multiple comorbidities, are continuously exposed to antibiotic therapy, and have a higher number of hospitalizations (4, 12, 20).

The most frequent comorbidities found were immunocompromise, arterial hypertension, diabetes mellitus, and vascular access exhaustion. The main factors associated with mortality were being ≥46 years of age, having diabetes mellitus and septic shock, requiring IMV, needing parenteral nutrition, exposure to CR-GNB, presenting BSI due to CRAB, and having received ineffective empirical treatment.

In the multivariate analysis, García-Rodríguez and Mariño-Callejo showed that severe sepsis/septic shock were predictive factors of ≤ 30-day mortality in patients with BSI due to MRD-GNB. And that adequate antimicrobial treatment was a protective factor (12). Also, Abubakar et al. found, in a multivariate regression analysis, that patients presenting with septic shock were a predictor of mortality, and that receiving active (appropriate) antibiotic treatment was protective, according to univariate analysis (21).

Greater vulnerability was found, as expected, by univariate analysis among CR-GNB patients compared with CS-GNB patients across risk factors and outcomes. Ineffective empirical treatment was found to be 30.2 times more frequent in CR-GNB patients, and ≤ 30-day mortality was 12.3 times more frequent in CR-GNB patients.

The main species responsible for GNB BSI were a small subset: *E. coli, K. pneumoniae, P. aeruginosa*, and *A. baumannii* (5). Abdel Hadi et al. reported in their cohort study that the predominant MDR-GNB pathogens were *E. coli, K. pneumoniae, Salmonella species*, and *P. aeruginosa* (4). Abubakar et al. report that *A. baumannii, Enterobacterales*, and *P. aeruginosa* are the most frequent MDR-GNB isolates among hospitalized patients in Malaysia. In our study, the most frequently isolated GNB were *A. baumannii, P. aeruginosa*, and *K. pneumoniae* (20). Also, in our study, 47.6% of the isolated GNB were susceptible to carbapenems, and 52.4% were resistant. In a multicenter retrospective study (19 Italian hospitals) that included 1,276 patients with GNB BSI, 56.7% were estimated to be susceptible to carbapenems and 43.3% were estimated to be resistant (18).

We observed lower mortality in patients with CS-GNB isolates, estimated at 6.7% (4/60), compared with a multicenter retrospective study reporting a mortality rate of 13.7% (99/723) for this subgroup (18). This is evident in our survival curves, where patients with CS-GNB isolation show a practically flat curve (Figure 3).

During follow-up (as part of the CS-GNB group), two outbreaks of *A. xylosoxidans* were documented in patients undergoing kidney transplantation, without serious problems. *A. xylosoxidans*, belonging to the *Alcaligenaceae* family, is usually considered an opportunistic and emerging pathogen in the hospital environment. It is a GNB, aerobic, and non-lactose fermenting. Outbreaks of nosocomial catheter-associated bacteremia due to *A. xylosoxidans* have been described, which were caused by contaminated environmental sources or devices, or by water faucets or hemodialysis systems, mainly in immunocompromised patients (21–23).

In patients with *P. aeruginosa* isolation, we estimated lower mortality in the CRPA group, 20.0% (2/10) vs. 32.8% (20/61) in the comparison group of Falcone et al. (18).

The mortality in the CRAB group was 50.0% (19/38), higher than that of this subgroup in the same comparison study (43.2%; 48/111) (18). In specific research on BSI by *A. baumannii*, Ngiam et al. reported a mortality of 73.3% for CRAB vs. 16.1% for carbapenem-susceptible *A. baumannii* (*p* < 0.001) (24).

The higher number of patients classified as CRAB is consistent with a previous study in our hospital for patients with nosocomial infectious ventriculitis caused by MDR-GNB associated with external ventricular drainage, where a higher percentage of isolation for *A. baumannii* was observed (40.6% of the total cerebrospinal fluid culture isolates) with 91.3% resistance to carbapenems (13).

In a meta-analysis, Du et al. reported factors associated with mortality in patients with BSI due to CRAB, including, as in the present study, ineffective empirical treatment, septic shock, total parenteral nutrition, and invasive mechanical ventilation (25).

In a retrospective cohort study, Son et al. evaluated risk factors for ≤ 30-day mortality in *A. baumannii* BSI and found septic shock and inappropriate antimicrobial therapy as independent risk factors for mortality (multivariate analysis). And, as an antibiotic strategy, they discovered that colistin combined with tigecycline or other antibiotics was significantly associated with lower mortality (after adjusting for confounding factors) (26). *A. baumannii* is responsible for a large number of nosocomial infections, with a significant capacity to survive in the hospital environment and to present antimicrobial resistance. Kumar et al., in a review, summarized the main resistance mechanisms of *A. baumannii*, including metallo-β-lactamase-type, OXA-type, and KPC-type carbapenemases, as well as efflux pumps responsible for multidrug resistance and aminoglycoside-modifying enzymes (27).

In patients with CRAB, we found 100% resistance to the entire spectrum of available older β-lactam antibiotics and quinolones, high resistance to aminoglycosides (65.8%), and resistance to tigecycline (44.7%).

A retrospective study that included a prognostic-matching analysis of BSI for *A. baumannii*, Wang et al., explains that providing appropriate empirical therapy to patients with septic shock, mechanical ventilation, and elevated SOFA scores can significantly improve survival (28). They point out that BSI preferentially affects critically ill patients. Of the patients with carbapenem-resistant *A. baumannii*, more than half presented with this condition; 23/38 (60.5%) were in an ICU, 33/38 (86.8%) required invasive mechanical ventilation, and 33/38 (86.8%) presented with septic shock; half died. Wang et al. also conclude that drug resistance in *A. baumannii* leads to inadequate empirical antibiotic therapy and is a direct predictor of mortality (28). These are also conclusive findings from our work. However, in clinical practice, we used this treatment due to the patient's condition. Previous studies explain that *A. baumannii* is very common in developing countries, so if there is no response or clinical improvement, colistin (polymyxin E) should be prescribed. Notably, colistin is also appropriate for definitive treatments (5, 24, 28, 29).

For the CRE group, a high mortality rate was observed, estimated at 58.3% (7/12), presenting one of the steepest survival curves in our work versus Falcone et al. (18) with an estimated mortality (KPC-type and, metallo-β-lactamase-type carbapenemases) of 40.0% (109/272). The high mortality rate found suggests that this group of GN bacilli should not be ignored. In particular, the CRE group stands out in the global panorama, with a significant increase in detection in the Asia Pacific, Europe, and Latin America regions (30).

In the Other Non-Fermenting GNB group, the high mortality rate was influenced by the *S. maltophilia* burden. This group showed the worst survival curve, but still outperformed the CRAB and CRE groups. *S. maltophilia*, an environmental aerobic GNB, acting mainly as an opportunistic pathogen, is of special interest. This is considered an essential nosocomial pathogen because it causes potentially fatal invasive infections. In our study, 4/5 patients with a positive culture for this GNB died (immunocompromised patients). According to the meta-analysis by Huang et al., there is insufficient data on an appropriate and specific therapy for *S. maltophilia* infection. Carbapenems are usually used empirically for any BSI due to GNB, which is inappropriate for *S. maltophilia* (intrinsically carbapenem-resistant GNB) (31). However, levofloxacin, trimethoprim/sulfamethoxazole, or the combined therapy of levofloxacin and trimethoprim/sulfamethoxazole can be used with favorable outcomes (30–33).

Among the *P. aeruginosa* isolates, six were classified as CS-GNB despite carbapenem resistance. Conversely, these same strains were found to be susceptible to cephalosporins (cefepime and ceftazidime) and piperacillin/tazobactam. Given this susceptibility, the reported resistance to carbapenems is likely due to porins or efflux pumps rather than carbapenemases. It has been suggested that alterations in the OprD porins responsible for the permeability of molecules such as carbapenems (through a gene produced by *P. aeruginosa*) and the presence of an active efflux pump are the main mechanisms associated with the resistance to carbapenems of this GNB. Furthermore, patients with these isolates were treated with cephalosporins with favorable outcomes (19, 34–37).

Along with the increase in the incidence of GNB bacteremia, the emergence of antimicrobial resistance in these bacilli has been reported, directly affecting treatment and increasing mortality. Inappropriate use of antibiotics has been reported in Malaysia, where up to 50% of prescriptions among hospitalized patients may not meet the specifications (5, 20). Although CR-GNB infections in the present study were associated with ineffective empirical treatment, the virulence of the isolated bacilli and delays in diagnosis should be considered. Notably, receiving ineffective treatment was an independent factor in mortality, according to the proportional HR model.

By obtaining an early diagnosis, it is easier to provide timely and appropriate treatment, which should be in accordance with the guidelines of the European Society of Clinical Microbiology (ESCMID) and the guidelines of the Infectious Diseases Society of America (IDSA), to improve prevention and stop the effects of drug resistance (3, 19, 27, 34, 38, 39). Furthermore, it is necessary to understand local epidemiological data and strengthen infection control programs.

## Limitations

International organizations closely monitor global fluctuations in behavior, as well as variations in the incidence, prevalence, and mortality of antimicrobial resistance, in which resistance to GNB plays a significant role. Despite limitations, primarily stemming from the retrospective nature of this study, the fact that the data correspond to a single hospital with complex characteristics, a lack of resources for performing high-tech microbiological typing (molecular typing and other methods), and potential operational differences with other research that affect the comparability of results, our work allowed us to epidemiologically profile what occurred during the study period. This profile reflects the complexity of managing human and material resources at our hospital, as well as its capacity to address emerging microorganisms.

During the study period, the hospital lacked the necessary technological resources to perform rapid, state-of-the-art tests for carbapenemase detection. This is consistent with the frequency with which patients receive ineffective empirical treatments.

In our hospital setting, therapeutic options for the treatment of BSI were limited during the study period, mainly due to the unavailability of colistin, ceftolozane/tazobactam, and ceftazidime/avibactam.

## Conclusions

Mortality in the bacteremia cohort of this study was associated with a high frequency of patients with CR-GNB, primarily due to CRAB, who received ineffective empirical treatment.

The information obtained through the follow-up and analysis of this cohort will undoubtedly improve medical care, accelerating the development of faster diagnostic tools and providing more effective treatments to our patients.

Since the data were collected over an entire year, a compilation of all BSI events, particularly those involving hospitalized patients due to GNB, is also presented, reflecting the complexity of these pathogens in the epidemiological and clinical landscape of our workplace, The Specialty Hospital, with 500 census beds.

Greater efforts are needed to improve epidemiological surveillance systems through Healthcare-Associated Infection and Antimicrobial Stewardship Committees. Furthermore, to adopt and implement universal healthcare measures, including different prevention packages (for each type of healthcare-associated infection) and other general measures such as staff training, water quality, environmental decontamination, antiseptic use, disinfection and sterilization, appropriate antibiotic use, use of isolation systems for proper patient management, and designated areas to improve medication preparation, among others. It is also necessary to promote education, monitoring, and feedback on guidelines for the prevention and control of healthcare-associated infections.

## Data Availability

The raw data supporting the conclusions of this article will be made available by the authors, without undue reservation.
